# Biological Solubilisation of Leather Industry Waste in Anaerobic Conditions: Effect of Chromium (III) Presence, Pre-Treatments and Temperature Strategies

**DOI:** 10.3390/ijms232113647

**Published:** 2022-11-07

**Authors:** Juana Fernández-Rodríguez, Beñat Lorea, Gustavo González-Gaitano

**Affiliations:** 1 Department of Chemistry, Instituto de Biodiversidad y Medioambiente (BIOMA), University of Navarra, 31080 Pamplona, Spain; 2 Department of Sciences, Institute for Advanced Materials and Mathematics (INAMAT2), Public University of Navarre (UPNA), Campus de Arrosadía, 31006 Pamplona, Spain

**Keywords:** anaerobic digestion, circular bio economy, chromium, leather, organic matter solubilisation, resource recovery, tannery waste

## Abstract

Collagen-based polymers and their blends have attracted considerable interest for new materials development due to their unique combination of biocompatibility, physical and mechanical properties and durability. Leather, a modified natural biopolymer made from animal rawhide and the first synthetic collagen-based polymer known since the dawn of civilization, combines all these features. Rawhide is transformed into leather by tanning, a process in which the collagen is cross-linked with different agents to make it stronger and more durable and to prevent its decay. Research on the development of environmentally friendly procedures and sustainable materials with higher efficiency and lower costs is a rapidly growing field, and leather industry is not an exemption. Chrome-tanned and vegetable-tanned (chromium-free) shavings from the leather industry present a high content of organic matter, yet they are considered recalcitrant waste to be degraded by microbiological processes like anaerobic digestion (AD), a solid technology to treat organic waste in a circular economy framework. In this technology however, the solubilisation of organic solid substrates is a significant challenge to improving the efficiency of the process. In this context, we have investigated the process of microbial decomposition of leather wastes from the tannery industry to search for the conditions that produce optimal solubilisation of organic matter. Chrome-tanned and chromium-free leather shavings were pre-treated and anaerobically digested under different temperature ranges (thermophilic–55 °C-, intermediate–42 °C- and mesophilic–35 °C) to evaluate the effect on the solubilisation of the organic matter of the wastes. The results showed that the presence of chromium significantly inhibited the solubilization (up to 60%) in the mesophilic and intermediate ranges; this is the fastest and most efficient solubilization reached under thermophilic conditions using the chromium-free leather shaving as substrates. The most suitable temperature for the solubilization was the thermophilic regime (55 °C) for both chromium-free and chrome-tanned shavings. No significant differences were observed in the thermophilic anaerobic digestion of chromium-free shavings when a pre-treatment was applied, since the solubilisation was already high without pre-treatment. However, the pre-treatments significantly improved the solubilisation in the mesophilic and intermediate configurations; the former pre-treatment was better suited in terms of performance and cost-effectiveness compared to the thermophilic range. Thus, the solubilisation of chromium-free tannery solid wastes can be significantly improved by applying appropriate pre-treatments at lower temperature ranges; this is of utter importance when optimizing anaerobic processes of recalcitrant organic wastes, with the added benefit of substantial energy savings in the scaling up of the process in an optimised circular economy scenario.

## 1. Introduction

Leather manufacturing and tannery are traditional industries with an extensive implantation that has been widely augmented in recent decades. It is an example of a circular economy since the 99% of leather production uses skins of animals from meat or milk manufacture [1]. Only the 20% by weight of the raw material is transformed into leather, the final product [2]. Tanned leather can be obtained from different sources, bovine tanning comprising 63% of the total production, followed by sheep (17%) and others, such as goat, pigs, rabbits and reptiles (20%) [3]. The main objective of the tanning process is the stabilization of collagen and other proteins present in animal skins by cross-linking agents, such as metals and/or polyphenols [4]. Chromium salts have special relevance, which have been used as an effective agent to stabilize the degradation of the material and which confers to leather valuable properties for different applications [5]. Tannins have a vegetal origin, and their mechanism consists of cross-linking the collagen of animal skins, which increases the resistance of the material to heat, water and the attack by microbes.

About 10% of the total weight of raw materials generates leather shavings, in the form of thin leather strips, as solid waste. Depending on the tanning process, these can contain chromium (in the wet blue process, which makes use of chromium salts) or be free from it (in the wet white process, which uses tannins). Both types represent a high-value, protein-based waste [6]. It has been estimated that about 75% of leather produced in the world is chrome-tanned [7]. Consequently, the effluents produced, both liquid and solid, include Cr(III)-containing wastes [8] that pose environment and human health risks due to the conversion of Cr(III) to Cr(VI) in the post-tanning treatments [9,10]. In this regard, to promote the reduction of Cr(VI), new research about advanced technology for wastewater treatment is being developed, especially based on photocatalysis [11], or adsorption/reduction with novel iron-based composites [12]. Actually, the environmental impact of the leather industry is a considerable concern [13,14]. In addition to chemical substances coming from tanning operations, a high organic load of byproducts, in the form of oils, fats and dyes, can be present in the final waste [15], which have to be considered for their environmental impact. Because of this, the deposit of the tanning wastes in landfills is discouraged and green alternatives should be promoted.

Different approaches have been proposed for the valorisation of leather shavings as an energetic source [16,17] or as building materials [18,19,20], among other alternatives. The high organic load of waste makes its valorisation possible using biotechnological strategies. However, in order to optimize the treatment and avoid operational problems, a proper physicochemical characterization of the waste is crucial to carry out its effective valorisation. Additionally, certain chemical compounds employed during the tanning operations can hinder the valorisation of the waste, a factor that should also be considered.

The valorisation and recuperation of waste from a previous industrial process is a key factor in the circular bioeconomy concept [21]. Anaerobic digestion (AD) is a microbiological process carried out on organic substrates and developed in the absence of oxygen that recovers biogas and a suitable substrate for composting. The raw biogas contains mainly of methane CH_4_ (40–75%) and carbon dioxide CO_2_ (15–60%). Minority components are water H_2_O (5–10%), hydrogen sulphide H_2_S (0.0005–2%) and other trace substances. The generated biogas can be stored, made available on demand and valorised (after its pre-treatment) as a renewable energy. The process of AD can be an attractive alternative in a biorefinery associated with the tannery sector, especially in the tannery wastewater line [22].

The global process of AD includes four linked metabolic phases, in which the products from the previous step are the substrates for the following: hydrolysis, acidogenesis, acetogenesis and methanogenesis [23]. AD is usually carried out from 30 to 65 °C, the temperature being the variable that conditions the evolution of microbial consortiums [24]. Traditionally, mesophilic (optimal 35 °C) and thermophilic (optimal 55 °C) have been the most-studied ranges. Thermophilic temperatures produce higher substrate degradation rates and increased microbial growth, thus shortening operation times. However, the mesophilic range reduces the potential inhibitory products, making it a more stable process with a lower energetic cost. Recently, an intermediate range (optimal 42 °C) that has shown a remarkable methane yield with lower energetic costs compared to the thermophilic one, is gaining interest [25,26].

AD has been used in the valorisation of organic animal waste [27]. In this sense, tannery solid waste could be a suitable substrate for anaerobic digestion, based on the anaerobic degradation of protein waste [28]. Several investigations have described relevant results on the AD of leather wastes, especially wastewater, most of them containing chromium. Zupančič and Jemec [29] obtained methane productions of 0.377 m^3^/kg from hide trimmings operating in thermophilic temperatures. Regarding tannery sludge, removals of 41–52% volatile solids (VS) and 71–77% methane yield were achieved in mesophilic temperatures [30]. The co-digestion processes (including mixtures of shavings and sludge) were also tackled at a lab and semi-pilot scale [31], with biogas yields in the 21–30 mL/kg VSS (volatile suspended solids) range, with as maximum methane content of 59% *v*/*v*, and a total organic carbon (TOC) reduction between 68 and 76%. These authors also reported a potential inhibitory effect of chromium on the AD process [31]. Additionally, some authors consider that hydrolysis is a limiting step of the AD [32]. In this regard, pre-treatments have shown to be highly effective in the global performance of the AD process, especially in the first step (hydrolysis of organic matter). Physicochemical pre-treatments of wastes are based on the combination of thermal cycles with chemical processes, which depend on the pursued objective. Especially interesting are those pre-treatments that promote the biomass solubilisation [33]. Hydrothermal Carbonization (HTC) is a thermochemical process used for the pre-treatment of wet biomass to enhance the mechanical dewatering, under the influence of subcritical water, at high temperatures (180–280 °C) and autogenous pressure [34]. This pre-treatment can be carried out with recalcitrant organic waste, improving the yield and methane potential of the substrates [35]. At lower temperatures, thermal hydrolysis (TH) consists of heating (120–180 °C) the organic substrate, typically for 15–40 min, which solubilizes complex organic materials, consequently improving the digestion [36,37,38]. The combination of TH with a chemical pre-treatment in tannery solid waste has also been demonstrated to improve the overall process with both chrome-tanned and chromium-free shavings [39].

Motivated by the scarce number of investigations on this relevant matter, the solubilisation of organic compounds from different tannery solid wastes through AD has been addressed in this work. The AD process has been carried out on the two typical types of tannery solid wastes (chrome-tanned and chromium-free leather shavings) under different temperature regimes: thermophilic (55 °C), intermediate (42 °C) and mesophilic (35 °C). A preceding physicochemical characterization of the leather shavings has been carried out, in order to establish correlations between the temperature at which the waste undergoes the pre-treatment and the solubilisation of organic matter. Based on this, different pre-treatments were applied to promote the solubilisation of waste, and the AD digestion processes was subsequently conducted on the previous temperature ranges using the pre-treated materials. The results here reported may provide useful in the estimation of the impact of temperature conditions and pre-treatments on the solubilisation of tannery solid waste, which is of interest in the development of more environmentally friendly processes in tannery industry.

## 2. Results and Discussion

The results and their discussion are presented under two sections, for the sake of clarity: untreated or pre-treated leather shavings. In each case, the temperature effects due to the presence of chromium (chrome-tanned waste, C) or otherwise (chromium-free, NC) on the anaerobic solubilisation processes. The thermal behaviour of the shavings is shown first, as a necessary preliminary step of the investigation.

### 2.1. Thermal Behaviour of the Leather Shavings

**Figure 1:**
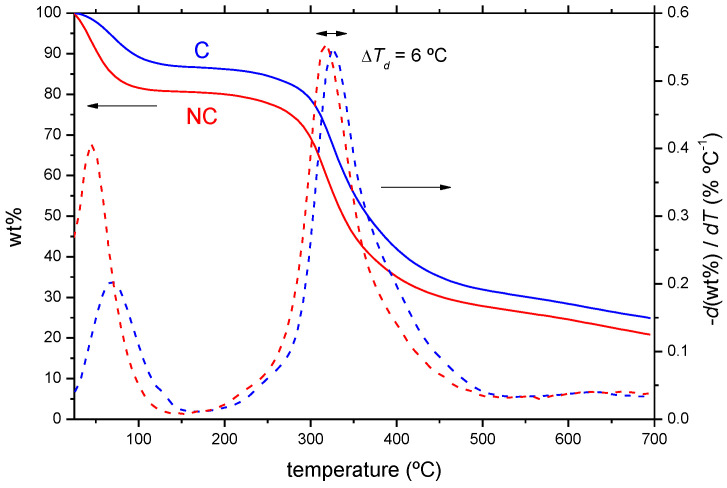
Thermogravimetric profiles (TGA and DTG) of chrome-tanned (C) and chromium-free (NC) leather wastes.

The thermal stability of the as-received shavings was studied by thermogravimetric analysis, TGA, and derivative thermogravimetry, DTG (Figure 1). Both types of leather waste display two mass losses, corresponding to water evaporation (50–150 °C) and to the pyrolysis of the organic matter (250–450 °C) (250–450 °C), with residues of 21 and 25% for NC and C shavings, respectively (26 and 29% in a dry basis). The water loss is slightly lower for the Cr(III) tanned waste (13% vs. 20%), most likely due to the different capacity of the shavings to trap moisture according to its texture. However, structural differences due to the presence of Cr(III) that make the waste less prone to the retention of water cannot be ruled out, since the water loss, that extends up to 150 °C, takes place at a considerably higher temperature (70 °C compared to 45 °C in C shavings). The C shavings are more resilient to degradation, which is reflected in a shift in temperature for the maximum speed of decomposition from 319 to 325 °C. This increase is ascribed to the stabilizing effect of the polychromium species that crosslink the collagen fibres. Marcilla et al., in a study of bovine and goat leathers [40], have reported a similar behaviour using vegetal, glutaraldehyde and chromium-based tanning.

Attenuated Total Reflectance Fourier Transformed Infrared (ATIR-FTIR) spectroscopy is a very convenient tool to investigate the secondary structure of proteins and the effects that variables, such as temperature, pH, solvent or denaturalizing agents may have on their structure. Supplementary Materials, Figure S1 shows the ATR-FTIR spectra at room temperature for both types of shavings (chrome-tanned and chromium-free), which reflects the high content of collagen present. The Amide I band of collagen (~1700 to 1600 cm^−1^) is the most intense and sensitive to the secondary protein structure, and it originates from the carbonyl stretching vibration and from both CN stretching and NH bending. It follows in intensity the Amide II band (~1600 to 1500 cm^−1^), which comprises peptide CN stretching and NH bending. The analysis of these bands usually presents the drawback of water absorption in this region, a hurdle that can be overcome by focusing on the less intense amide III band (~1310 to 1175 cm^−1^), associated with CN stretching and NH bending vibrations, CC stretching and CH bending. Despite its lower intensity, this band is very sensitive to protein secondary structure and it is not affected by the OH bending mode of water [41]. The comparison between the spectra of the two types of shavings shows differences in intensity in the 2700–3600 cm^−1^ region, corresponding to amide A and B bands and to the OH stretching vibration of water. These bands are more intense for the C samples due to the higher humidity content, in line with the TGA results. The amide I and II bands are similar in both types of shavings, while amide III band shows differences in the relative intensities of the signals at 1227 and 1165 cm^−1^. The presence of chromium entails interactions with aspartic and glutamic residues in the collagen helix that affect its intramolecular structure, changes that can be detected by FTIR, despite the relatively low content of metal (1.8%, as determined by FAAS).

**Figure 2:**
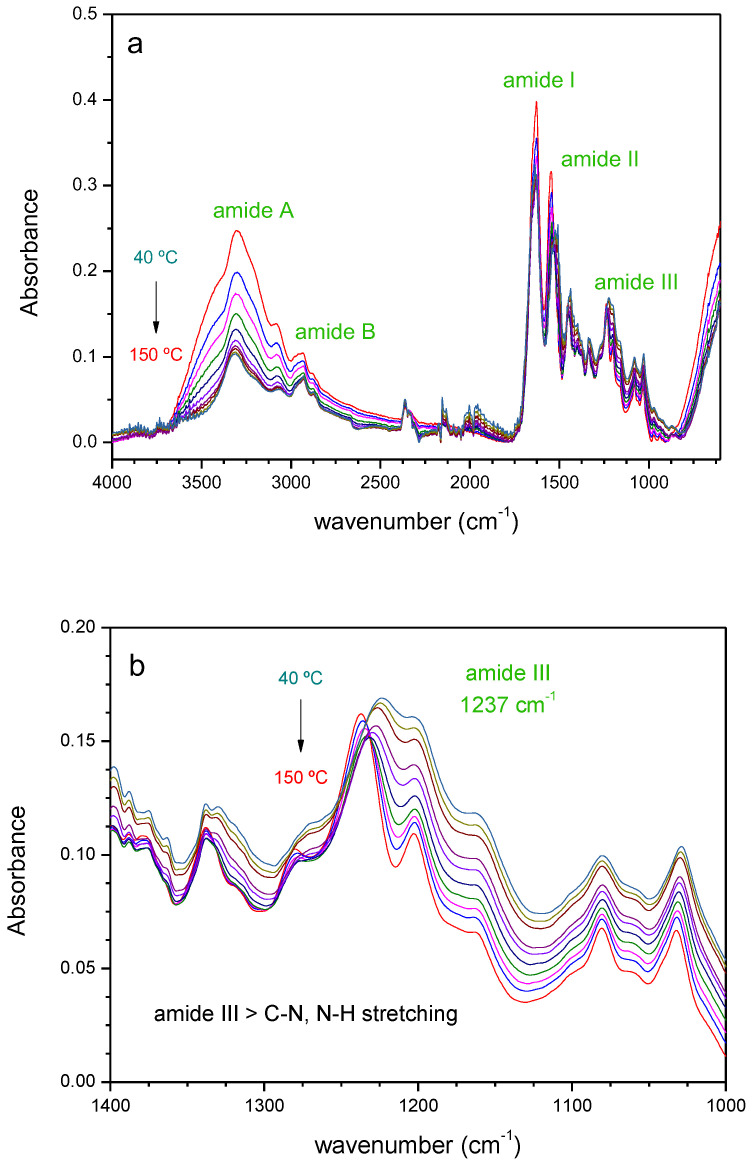
(**a**) ATR-FTIR spectra of Cr(III)-free leather waste as a function of the temperature; and (**b**) zoomed-in view of the amide III zone of the FTIR spectra of Cr(III)-free leather waste as a function of the temperature.

**Figure 3:**
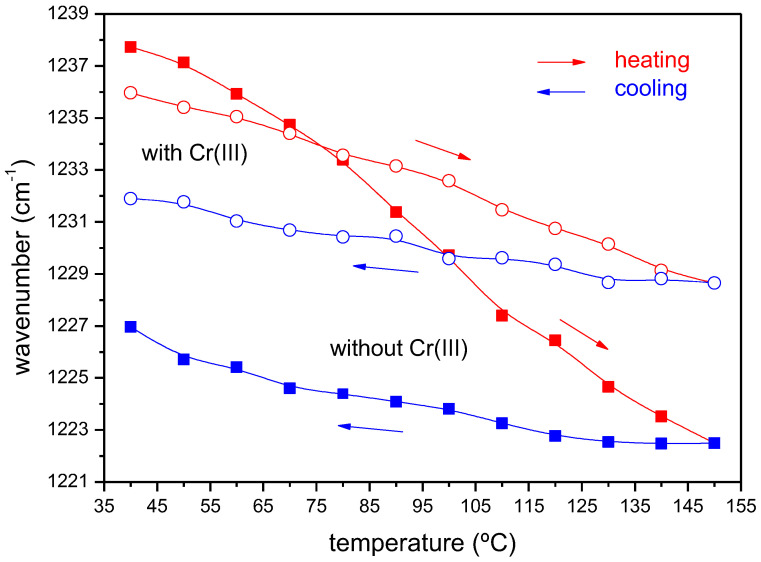
Shift of the amide III band as a function of the temperature (heating and cooling runs), showing the structural hysteresis.

The changes occurring in the ATR-FTIR spectra with the temperature can provide valuable information on the effects of a thermal pre-treatment on the structural features of the material. The denaturalization of type I collagen has been studied by FTIR in the range 20 °C to 80 °C; the changes observed in the Amide III band are ascribed to the rupture of the intermolecular bonds that connect the amino acidic residues of the protein [41]. Other studies have used this spectroscopy to determine the thermal degradation stages at higher temperatures in goat and sheep tanned leathers [42]. Figure 2 shows the vibrational spectra of the chromium-free leather shavings at different temperatures. The absence of new bands in the spectrum (or the disappearance of existent ones) even though heating up to 150 °C (close to the start of the thermal decomposition, Figure 2a) indicate the lack of relevant chemical changes occurring in this temperature range, consistent with the highly stable structure of the collagen helix. Focusing on the region at high wavenumbers of the mid-IR spectrum, the intensity of the bands at 3300 cm^−1^ diminish with the temperature until reaching a constant value at 150 °C. This zone contains an important contribution of the stretching vibration of OH groups in water, and the intensity reduction observed is mainly due to the humidity loss of the shavings as the temperature increases. In the fingerprint region (Figure 2b), the most relevant changes occur in the amide bands. Amide I and Amide II bands show a progressive reduction in intensity, in line with the loss in absorption of the bending mode of water overlapping this region. The presence of an isosbestic point at 1530 cm^−1^ suggests the presence of two different forms of the biopolymer, with a blue shift and intensity reduction of the signal at 1546 cm^−1^ accompanied by the increase of the 1508 cm^−1^ band. The less intense Amide III (1237 cm^−1^) shows an initial absorbance reduction accompanied by a blue-shift. Interestingly, if the sample is cooled down from 150 °C, the amide III band red shifts, but without reaching the initial value, indicating a recovery of the native structure that is only partial (structural hysteresis) that strongly depends on the presence of Cr(III). The wavenumber of the band along the heating and cooling cycles has been plotted in Figure 3 for both types of waste. The C wastes undergo a much lower degree of hysteresis compared to the Cr(III) free samples, as an indication that the collagen structure is restored to a higher extent. The chrome-tanned wastes again show a higher resilience upon heating, in this case at temperatures that do not involve thermal decomposition. According to Hassan, 130 °C would be a critical temperature at which the proteins of leather begin to unfold and degrade [42]. When conducting our experiments on NC shavings heating up to 120 °C, the “recovery” of the collagen structure, as estimated from the wavenumber shift of the amide III band along the thermal cycle, is 8.5 cm^−1^, compared to 10.7 cm^−1^ at 150 °C. A rough calculation would indicate an 85% denaturation degree, assuming that the collagen is fully denaturised at 150 °C. In terms of a thermal pre-treatment (or its combination with a chemical one), and for practical purposes of waste treatment on a large scale, a good compromise between the temperature applied and the degree of collagen denaturalisation would be some value at around 120 °C, which could be reduced in the case of vegetable-tanned leather wastes. Based on these findings, the study of solubilisation by anaerobic digestion was carried out at different temperatures and pre-treatments, as follows:

### 2.2. Solubilisation of Chrome-Tanned and Chromium-Free Leather Shavings: Effect of Temperature

Three thermal regimes were assessed to investigate the solubilisation of organic compounds from the leather shavings. The reactors were running for 55 days to guarantee the solubilisation step, and different parameters were analysed to study the performance under the selected experimental conditions. The following results have been calculated in all cases by subtracting the influence of the inoculum at different temperatures (35, 42 and 55 °C), as a blank. No valuable biogas production was recovered with the experimental set-up used, most likely due to the recalcitrant nature of these wastes, and that the investigation has focused on the solubilisation of organic matter from the leather shavings to the liquid phase. 

#### 2.2.1. Study of pH Evolution

The pH is an indicator of the stability and evolution of AD, since the different microbiological groups involved work at optimal and inhibitor pH values. The evolution of pH in the reactors at the different temperatures is shown in Supplementary Materials, Figure S2. It can be highlighted that the pH is acidic, corresponding to hydrolysis and acidogenesis phases (5.5–7.0). The elapsed time until reaching the pH stability varied according to the substrate composition and temperature operation. The shortest run lasted 25 days under thermophilic (55NC) conditions. The mesophilic (35NC) and intermediate temperatures (42NC) of the samples without chromium required more time until pH stability (around 35 days). Similar times were observed for all the chrome-containing samples (55C, 42C and 35C). It is worth mentioning that the stabilized pH for 55NC was higher than that of 55C (8.5 versus 7.5), which could be related to a higher stability [43], since pH under 8.5 prevents the methanogenesis inhibition [44].

#### 2.2.2. Volatile Solids (VS) Evolution

The VS production over time followed different patterns depending on the substrates and temperatures. VS concentration remained constant until day 25 in the mesophilic range and until day 37 in the thermophilic and intermediate ranges, in which the concentration started to decrease in the case of C shavings (Supplementary Materials, Figure S3a). In NC wastes, the faster removal was under the thermophilic range, around 15 days, while at the mesophilic and intermediate temperatures, the consumption of VS started around 30–35 days (Supplementary Materials, Figure S3b).

When comparing both substrates, it is observed that the VS removal was higher in the chrome-tanned shavings (28.3% in 42C and 9.6% in 42NC; 49.9% in 35C and 21.8% in 35NC), except in the thermophilic temperature and chromium-free shavings (Table 1). The maximum removal was observed in 55NC, yielding 73.8% versus 16.8% at 55C.

#### 2.2.3. Soluble Chemical Oxygen Demand (COD_s_) and Dissolved Organic Carbon (DOC)

**Figure 4:**
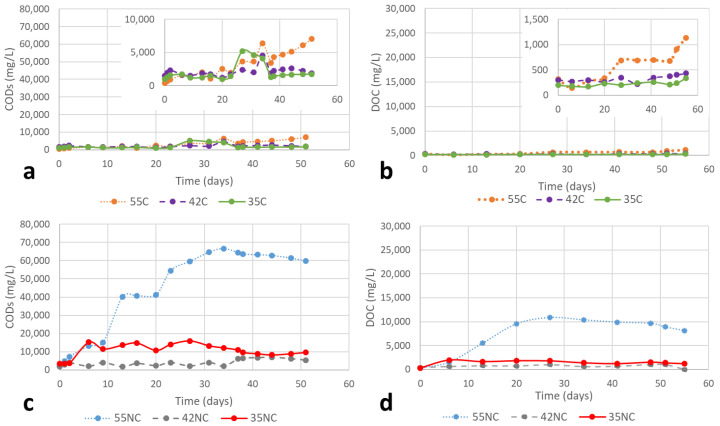
Evolution of organic matter solubilisation at thermophilic (55 °C), intermediate (42 °C) and mesophilic (35 °C) temperature regimes: (**a**) COD_s_ from chrome-tanned (C); (**b**) DOC from chrome-tanned (C); (**c**) COD_s_ from chromium-free (NC); and (**d**) DOC from chromium-free (NC) leather shavings.

The solubilisation of organic matter was quantified as soluble COD_s_ (Figure 4a,b) and DOC (Figure 4c,d; and Table 1). A fraction of organic substrates was solubilized for all the experimental setups. With respect to Cr(III)-containing substrates, in general, no significant solubilisation was observed. Comparing the temperature conditions, the best performance was observed with the highest temperature (55C). In thermophilic regime, the increase of COD_s_ concentration in the liquid started from day 20. The solubilisation continued at the end of the experiment (day 55) since the organic matter was still released to the medium. In the case of NC shavings, the best conditions were by far the thermophilic temperature. The presence of Cr(III) in the substrate clearly limits the solubilisation of the waste, resulting in lower values in all the cases (61.4% in the thermophilic range; 66.9% in the intermediate range and 36.6% in the mesophilic range). The DOC followed a similar trend to the COD_s_ (Table 1). The cross-linking action of Cr(III) that provides stability to the collagen fibres may be in the origin of this behaviour.

### 2.3. Solubilisation of Chrome-Tanned and Chromium-Free Leather Shavings: Effect of Temperature and Pre-Treatment

The physicochemical pre-treatments were applied to promote the maximum solubilisation of the tannery shavings. Due to the nature of the waste, the pre-treatment proposed by Dhayalan et al. [39] was carried out, consisting of a combination of physical and chemical pre-treatments, which distinguishes the presence or absence of chromium in the waste, as described in the *Material and Methods* section. The first stage (physical pre-treatment) includes a thermal period at 120 °C, a temperature that is consistent with the results obtained from the FTIR investigation described in Section 2.1 and proposed in the bibliography as the best approach [37]. Likewise, according to the results described in Section 2.2, the solubilisation experiments were extended up to 35 days.

#### 2.3.1. Study of pH Evolution 

The evolution of pH was conditioned by the presence of Cr(III) in the waste. After 35 days, the pH was between 6.5 and 7.0 in the substrates containing Cr(III) and in the range 7.0 and 8.0 in those without Cr(III) (Supplementary Materials, Figure S4). Since the increase of pH can be related with a higher stability, the solubilisation process would be favoured in the absence of Cr(III). 

#### 2.3.2. Volatile Solids (VS) Evolution

The removal of VS was similar in both substrates, in the range 56–62% (Supplementary Materials, Figure S5 and Table S2), although the evolution was different to the general trend at the intermediate temperature and in the presence of Cr(III) (18%). The maximum consumption of VS extended until day 10 in all cases except in 42C-P (day 25). According to our findings (Section 2.2.2), the pre-treatments shorten the time of degradation (10 days versus 15–55 days without pre-treatment). On the other hand, the consumption of VS was higher in the pre-treated samples: 56–62% versus 6–15% in the non-pre-treated wastes. 

#### 2.3.3. Soluble Chemical Oxygen Demand (COD_s_) and Dissolved Organic Carbon (DOC)

**Figure 5:**
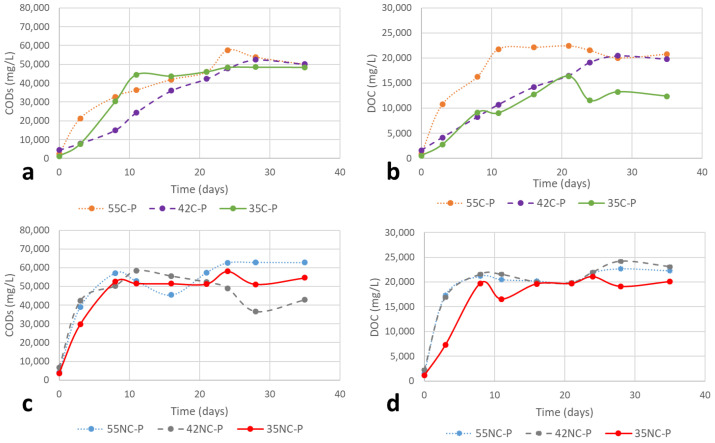
Evolution of organic matter solubilisation at thermophilic (55 °C), intermediate (42 °C) and mesophilic (35 °C) temperature regimes: (**a**) COD_s_ from pre-treated chrome-tanned (C-P); (**b**) DOC from pre-treated chrome-tanned (C-P); (**c**) COD_s_ from pre-treated chromium-free (NC-P); and (**d**) DOC from pre-treated chromium-free (NC-P) leather shavings.

The solubilisation of organic matter showed different trends in the pre-treated substrates depending on the temperature and presence or absence of Cr(III). Regarding the COD_s_ in chrome-tanned shavings (Figure 5a), the solubilisation in the thermophilic regime started from the very beginning until day 22, and the intermediate temperature showed a progressive increase until day 26, while the mesophilic regime produced 90% of the solubilisation in the first 11 days. Regarding the evolution of COD_s_ in chromium-free leather shavings (Figure 5b), the trend is similar at the beginning of the process for all the temperatures (the solubilisation occurred during the first 8 days). Nevertheless, at the end of the run (35 days) some differences were observed. The thermophilic regime was the temperature at which the highest concentrations of soluble organic matter were measured; the intermediate regime, the lowest. The DOC showed a similar trend of COD_s_, with a faster solubilisation in the case of chromium-free shavings (8 days) versus chrome-tanned (12–23 days) (Figure 5c,d).

Regarding the consumption of VS and TS (Table 1 and Table 2), it results higher in all cases at the pre-treated conditions (300–800%) over the not pre-treated waste, except in 55NC shavings in the thermophilic temperature. Under these conditions (no pre-treated substrate), the consumption of total solids was the highest (65.5%) and the pre-treatment did not improve the process.

**Figure 6:**
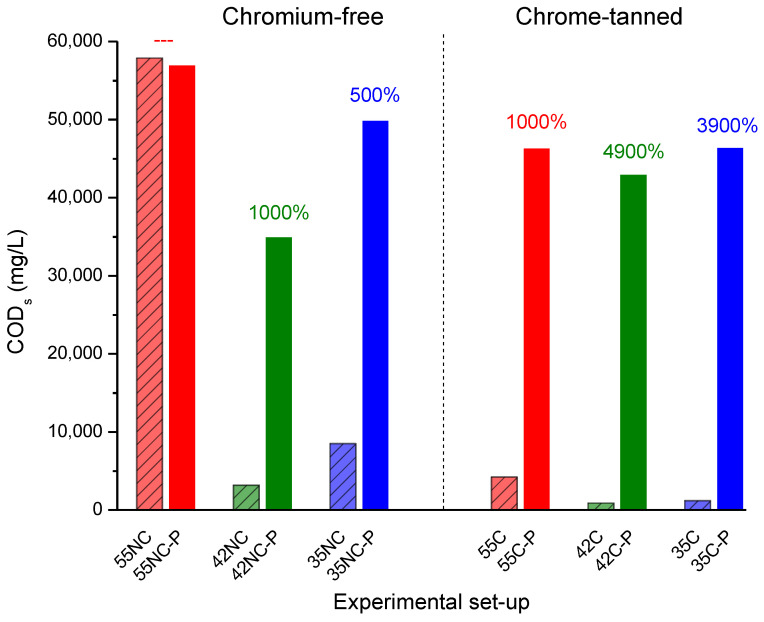
Solubilisation of the leather shavings expressed as CODs (mg/L) at different temperatures (55, 42, 35 °C). Not pre-treated (-); pre-treated (P); with Cr(III) (C) and without Cr(III) (NC). On top of bars, increments over the not pre-treated substrates.

The increment of DOC (the solubilisation of the organic solid waste) was remarkable in all the experimental conditions when the pre-treatment was applied (500–4,900%). These results agree with reported studies on the effect of thermal pre-treatment on the solubilisation of organic compounds [37]. However, the lowest DOC enhancement was measured for 55NC-P, since DOC was the highest recorded in 55NC (the process without pre-treatment). In this regard, a significant improvement in solubilisation by applying pre-treatments related to CODs is observed under all the conditions studied, except in chromium-free shavings and the thermophilic regime, 55NC (Figure 6). In the thermophilic temperature regime, the solubilisation was so high that the pre-treatment did not improve the initial results without pre-treatment with the different studied parameters.

Thus, the maximum percentages of solubilisation were found, in general, at lower temperature ranges: mesophilic (35 °C) and intermediate (42 °C). In all cases, the impact of pre-treatments on the solubilisation was higher in the substrate containing chromium. In this regard, in 55NC shavings, no improvement was recorded after pre-treatment; however, 1000% approx. was reached in the substrate with chromium (55C). Regarding the intermediate temperature, the solubilisation from the chromium-tanned shavings was 4.9 times higher after pre-treatment (1000 versus 4900%). In the mesophilic range, the increment of DOC was 500% in the NC shavings, compared to 3900% in the C shavings. These results are especially relevant since the wet blue process continues being the most common process applied in the leather industry (about 75%, [7]).

## 3. Materials and Methods

### 3.1. Substrates 

**Figure 7:**
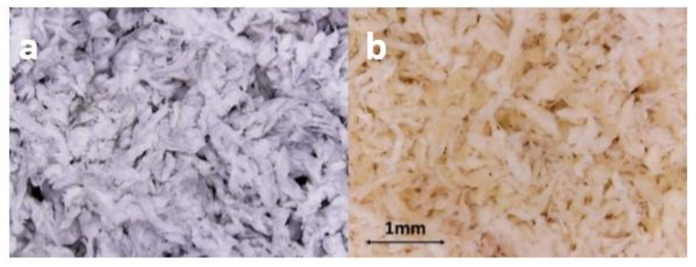
Micrographs of the leather shavings used: (**a**) Chrome-tanned (C), tanned from the wet blue industrial process; and (**b**) chromium-free (NC), from the wet white industrial process.

Bovine leather shavings were provided from Tenerías Omega SA (Villatuerta, Navarra, Spain). Two types of shavings were collected: Chrome-tanned (C) from the wet blue industrial process, and Chromium-free (NC) shavings from the wet white industrial process (Figure 7). The inoculum for the AD was obtained from an anaerobic mesophilic digester operating in an Arazuri wastewater treatment plant (Navarra, Spain). The inoculum was acclimated for the experimental temperature conditions over a period of 72 h with a source of glucose.

### 3.2. Experimental Set-Up

**Figure 8:**
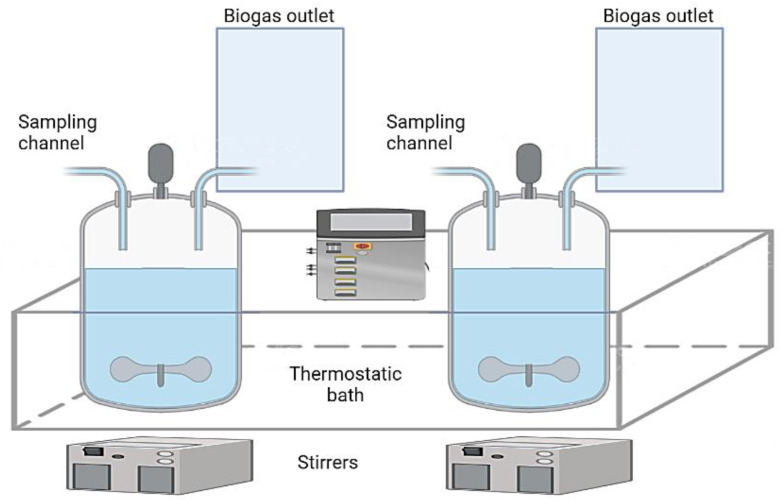
Scheme of the experimental setup for the AD.

The solubilisation of the leather shavings was carried out in anaerobic conditions according to Holliger et al. [45]. Continuously-stirred tank reactors, CSTR, in batch mode (1.0L total volume and 0.8L working volume) were used (Figure 8). The biogas was collected from the outlet in 5 L-fluorinated ethylene propylene bags (brand SKC). The reactors were immersed in a thermostatic bath to control the temperature. The wet range, with 6% total solids (TS), was chosen, with the inoculum representing one third of the reactor’s working volume (0.27 L). A nutrient solution (0.05 L), prepared according to de Diego-Díaz et al. [46], was added to each reactor to avoid nutritional deficiencies. The process was conducted at different temperature regimes: the mesophilic (35 °C), intermediate (42 °C), and thermophilic (55 °C) temperatures. Following the studies of Dhayalan et al. [39], a pre-treatment was applied, depending on the presence or absence of Cr(III) in the leather solid waste. The process consisted in the removal of tanning agents from the shavings, based in a thermal cycle (120 °C, 15 min in autoclave) followed by a chemical treatment. The detailed chemical pre-treatments, depending on the type of waste, were:

– C leather shavings: 2% oxalic acid and 10 mM ethylenediaminetetraacetic acid (EDTA), 2 h stirring with 200% water at room temperature.
– NC leather shavings: 1.5% sodium borate (borax) and 0.5% sodium sulphate, 2 h stirring with 100% water at room temperature.
– The description of the experimental set-up, and acronyms used for the different conditions and corresponding operational parameters, is summarized in Table 3.

### 3.3. Characterization Methods

**Atomic spectroscopy.** Flame atomic absorption spectrometry (FAAS) was employed to determine the content of chromium in the shavings, using a Perkin Elmer Analyst 800 atomic absorption spectrophotometer with an air–acetylene flame. Sample digestion was carried out following the protocol set out in the literature [47]. After digestion, the content of metal was obtained through calibration with a series of standard solutions in the linear range (5.0 mg/L), using the 357.9 nm wavelength of a Cr hollow-cathode lamp (0.7 nm slit width).

**Infrared spectroscopy.** Attenuated Total Reflectance Fourier Transform Infrared Spectroscopy (ATR-FTIR) was used to evaluate the structural response of the waste under heating and cooling cycles. The spectra were recorded in the 600–4000 cm^−1^ range on a Shimadzu IRAffinity-1S spectrometer (Kyoto, Japan) equipped with a diamond-window, temperature-controlled ATR accessory (Golden-Gate, from Specac). Thirty-two scans per spectrum were collected with a 4 cm^−1^ resolution, in temperature cycles that varied from 40° to 150 °C and downwards. The as-received shavings were pressed directly onto the ATR crystal with a clamp, after running the background spectrum at the beginning of the thermal cycle.

**Thermal analysis.** The thermal degradation of the wastes was performed by thermogravimetric analysis (TGA). Around 10 mg of the waste (sizes in the range of 2.5–4 mm) were introduced in Al_2_O_3_ crucibles and the thermograms recorded in a TG-sDTA 851 Mettler-Toledo thermal analyzer (Columbus, OH, USA), using a heating rate of 10 °C min^−1^, nitrogen atmosphere and a temperature range from 25 to 700 °C. The derivative thermogravimetric (DTG) curves of the evolution of sample weight with the temperature were obtained with the implemented software, STARTe 9.20. 

**Solubilisation studies under anaerobic conditions.** The analysed parameters related to the monitoring of the process were total solids (TS), volatile solids (VS), soluble chemical oxygen demand (COD_s_) and dissolved organic carbon (DOC). TS and VS are based on gravimetric methods. COD_s_ was determined by acid digestion and colorimetric analysis, using an Agilent 8453 UV-vis spectrometer (Santa Clara, CA, USA). DOC, based on the thermal oxidation to CO_2_, was carried out with a TOC-L Shimadzu apparatus (Kyoto, Japan), equipped with a non-dispersive infrared detector, NDIR. Biogas production content was determined by bubbling the biogas through a gasometer containing an alkaline solution [45] (Holliger et al., 2016). Additionally, a Geotech Biogas5000 biogas analyser (Wuhan, 430205, China) was used to quantify the composition of biogas. The analytical methods for the solubilisation performance were carried out according to standardized procedures [48]. 

### 3.4. Substrate Characterization

The waste and the inoculum were previously characterized to set the initial conditions and performance of the processes. Supplementary Materials, Table S1 shows the initial characterization of the solid content of the substrates and the inoculum as Total Solid (TS), Volatile Solid (VS) and organic content over the dry weight, in mass percentage. The TS is higher in chromium-free leather shavings compared to chrome-tanned ones (51.2% vs. 45.2), and the proportion of organic content is 98 and 94%, respectively. The particle size distribution (PSD) was measured by sieving, and a size range of 2.5–4 mm was selected for the characterization techniques and pre-treatments. Regarding the Cr(III) content, the average value of triplicate measurements was 18 mg/g of waste (1.8% by mass), typical of chrome-tanned leathers [49,50].

Before starting the experiments in the anaerobic reactors, the different substrates were dried (75 °C during 24 h) and ground in a blade mill, to assure their homogenisation. The final size of the particles was in the 10–220 μm range, as determined by optical microscopy and image analysis.

## 4. Conclusions

The motivation of this work has been to address the challenge of the solubilisation of solid organic wastes, based on collagen polymers, from the leather industry, which uses tanning processes that involve chromium and other hazardous chemicals. Our strategy has been to first study the effect of temperature on leather shavings by spectroscopic and thermal methods to ascertain the best conditions to optimize the solubilisation of the organic matter of the waste and their further application in the anaerobic digestion processes.

The study by ATR-FTIR of the as-received tannery waste reveals different thermal hysteresis behaviour depending on the presence or absence of Cr(III), as deduced from the temperature dependence of the amide B band of the collagen present in the hide. The chrome-tanned waste shows higher resilience upon heating and higher stability against thermal decomposition. In terms of a thermal pre-treatment, a good compromise between temperature and degree of collagen denaturalization can be achieved with 120 °C, although this value could be lowered in the case of chromium-free waste.

The solubilisation of leather shavings in anaerobic conditions was influenced by the presence of Cr(III), being substantially higher in the case of chromium-free shavings. Regarding the effect of the temperature, the maximum degree of solubilisation was achieved with the thermophilic regime (55 °C), followed by the mesophilic (35 °C) and the intermediate temperature (42 °C). The maximum solubilisation was obtained in the thermophilic chromium-free shavings reactors (55NC).

Upon pre-treatment, positive relevant results in terms of solubilisation were obtained with both types of substrates (C and NC), but especially with the chrome-tanned shavings, in line with the low solubilisation achieved with this type of waste when the pre-treatment is not applied. On the other hand, the solubilisation depended on the thermal conditions applied. On chromium-free pre-treated shavings, no significant differences were observed in the thermophilic range. The impact of the pre-treatment in the other thermal ranges, intermediate and mesophilic, was considerable. This is linked with the low solubilisation achieved with this type of waste in the thermophilic range without the pre-treatment. 

In summary, the solubilisation of chromium-free shavings is highest in thermophilic (55 °C), but significant improvements can be achieved using appropriate pre-treatments in all the temperature ranges when the waste contains chromium. The pre-treatment at the lowest temperatures (35 and 42 °C) on the chrome-tanned shavings delivered the highest solubilisation. The relevance of these results is that they demonstrate the substantial improvement in the solubilisation of organic matter from chrome-tanned leather shavings, which constitute the majority of waste in the leather industry, while the optimised conditions may lead to considerable energy savings on an industrial scale. Hence, the pre-treatment is a key factor in order to accomplish the solubilisation of tannery solid waste in further AD processes, promoting the circular economy of this recalcitrant organic waste.

## Figures and Tables

**Table 1:** Degradation of total (TS) and volatile solid (VS) and solubilisation of chrome-tanned (C) and chromium-free (NC) leather shavings at different temperatures, after 55 days.

Reactor	T (°C)	Substrate	TS (%)	VS (%) **	COD_s_ (mgO_2_/L) *	COD_s_ (%)	DOC (mg/L) *	DOC (%)
55C	55	Cr(III)	−10.0	−5.9	4213	971	484	152
55NC		Cr(III)-free	−65.5	−73.4	59708	25211	9294	2820
42C	42	Cr(III)	−16.2	−12.6	867	56	50	16
42NC		Cr(III)-free	−8.0	−8.6	3167	170	323	121
35C	35	Cr(III)	−32.2	−14.6	1470	144	60	30
35NC		Cr(III)-free	−9.4	−10.6	7741	228	1101	352

* Calculated from average values in stability phase (from 20 days) after subtracting the initial value (t = 0), divided by the initial value and multiplied per 100; ** % of volatile solids on the total weight (including water and total solids).

**Table 2:** Degradation of solid (TS) and volatile solid (VS) and solubilisation of pre-treated leather shavings, chrome-tanned (C-P) and chromium-free (NC-P), at different temperatures, after 35 days.

Reactor	T (°C)	Substrate	TS (%)	VS (%) **	COD_s_ (mgO_2_/L) *	COD_s_ (%)	DOC (mg/L) *	DOC (%)
55C-P	55	Cr(III) pre-treated	−36.3	−56.5	49,520	2109	19,888	2234
55NC-P		Cr(III)-free pre-treated	−50.7	−56.6	58,768	1446	20,619	1256
42C-P	42	Cr(III) pre-treated	−19.1	−18.4	46,729	1030	18,189	1144
42NC-P		Cr(III)-free pre-treated	−58.1	−61.1	33,029	492	20,858	927
35C-P	35	Cr(III) pre-treated	−31.4	−57.8	47,200	3762	11,849	2154
35NC-P		Cr(III)-free pre-treated	−57.5	−62.0	49,225	1342	18,917	1653

* Calculated from average values in the stability phase (from 15 days) after subtracting the initial value (t = 0), divided by the initial value and multiplied by 100; ** % of volatile volids on the total weight (including water and total solids).

**Table 3:** Description of the experimental setup.

Nomenclature	Substrate	Inoculum	T (°C)	Pre-Treatment
35	--	Yes	35	-
35NC	Chromium-free leather shavings	Yes	35	-
35C	Chrome-tanned leather shavings	Yes	35	-
42	--	Yes	42	-
42NC	Chromium-free leather shavings	Yes	42	-
42C	Chrome-tanned leather shavings	Yes	42	-
55	--	Yes	55	-
55NC	Chromium-free leather shavings	Yes	55	-
55C	Chrome-tanned leather shavings	Yes	55	-
35NC-P	Chromium-free leather shavings	Yes	35	***Thermal*** 120 °C, 15 min ***Chemical*** 1.5% sodium borate 0.5% sodium sulphate 25 °C, 120 min
35C-P	Chrome-tanned leather shavings	Yes	35	***Thermal*** 120 °C, 15 min ***Chemical*** 2% oxalic acid 10 mM EDTA 25 °C, 120 min
42NC-P	Chromium-free leather shavings	Yes	42	***Thermal*** 120 °C, 15 min ***Chemical*** 1.5% sodium borate 0.5% sodium sulphate 25 °C, 120 min
42C-P	Chrome-tanned leather shavings	Yes	42	***Thermal*** 120 °C 15 min ***Chemical*** 2% oxalic acid 10 mM EDTA 25 °C, 120 min
55NC-P	Chromium-free leather shavings	Yes	55	***Thermal*** 120 °C, 15 min ***Chemical*** 1.5% sodium borate 0.5% sodium sulphate 25 °C, 120 min
55C-P	Chrome-tanned leather shavings	Yes	55	***Thermal*** 120 °C, 15 min ***Chemical*** 2% oxalic acid 10 mM EDTA 25 °C, 120 min

## Data Availability

The datasets generated and/or analyzed are available from the corresponding author upon request.

## Supplementary Materials

The supporting information can be downloaded at: https://www.mdpi.com/article/10.3390/ijms232113647/s1.
