# Supplementary material for: Biological Solubilisation of Leather Industry Waste in Anaerobic Conditions: Effect of Chromium (III) Presence, Pre-Treatments and Temperature Strategies
Source: Int J Mol Sci. 2022 Nov 7;23(21):13647. doi: 10.3390/ijms232113647 (PMC9659162; doi:10.3390/ijms232113647)
Supplement: Supplementary file 1 [file ijms-23-13647-s001.zip › ijms-2026073-supplementary.pdf]

International Journal of
Molecular Sciences

MDPI

Supplementary Data

Biological solubilisation of leather industry wastes in anaerobic conditions: Effect of Chromium (III) presence, pre-treatments and temperature strategies

Juana Fernández-Rodríguez [1,*], Beñat Lorea [2] and Gustavo González-Gaitano [1,*]

[1] Department of Chemistry, Instituto de Biodiversidad y Medioambiente (BIOMA), University of Navarra, 31080 Pamplona, Spain
[2] Institute for Advanced Materials and Mathematics (INAMAT2), Department of Sciences, Public University of Navarre (UPNA), Campus de Arrosadía, 31006 Pamplona, Spain
* Correspondence: juanafernandez@unav.es (J.F.-R.); gaitano@unav.es (G.G.-G.)

Table S1. Basic characterization of tannery solid wastes and inoculum.

	TS (%)	VS (%) *	% Organic content **	Cr (III) (mg/g)
**Inoculum**	3.0 ± 0.3	1.9 ± 0.4	61.3± 0.9	-
**Chrome-tanned leather shavings**	45.2 ± 1.0	42.5 ± 1.5	94.0± 1.9	18± 0.6
**Chromium-free leather shavings**	51.2 ± 0.8	50.6 ± 1.4	98.8± 1.1	-
**Particle Size Distribution (PSD)**				
**Particle size (mm)**	>4	2.5-4	1.25-2.5	<1.25
**% (mass)**	40.9	27.8	6.2	25.0

* Over the total weight (wet weight). ** Over the dry weight (TS)

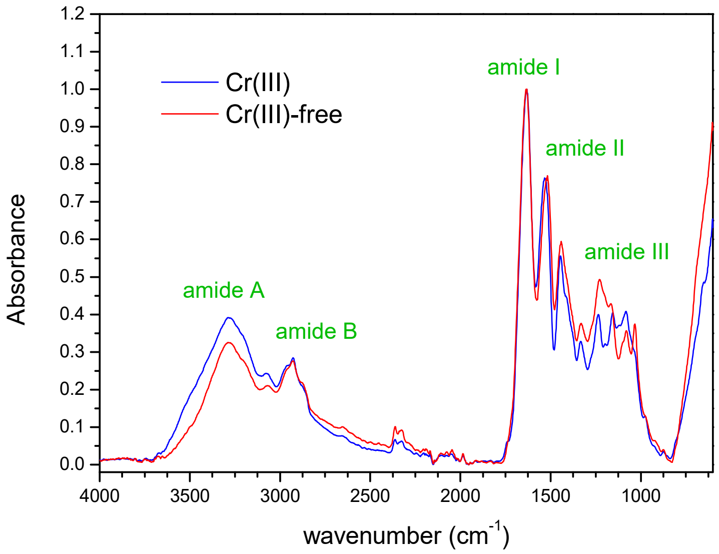

Figure S1. Normalized ATR-FTIR spectra of the as-received leather shavings.

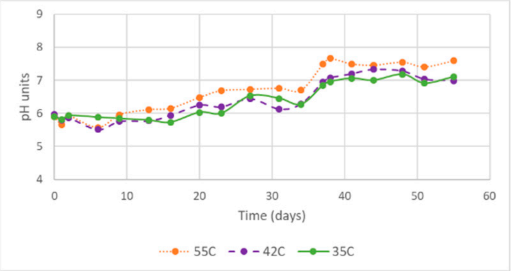

(**a**)

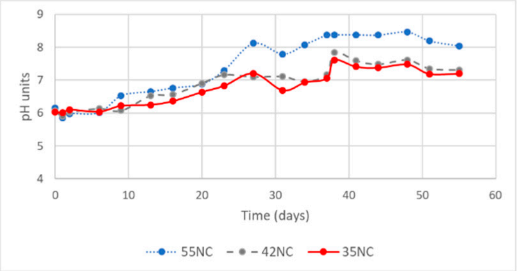

(**b**)

Figure S2. Evolution of pH at thermophilic (55ºC), intermediate (42ºC) and mesophilic (35ºC) regimes: a) chrome-tanned (C); b) chromium-free (NC) leather shavings.

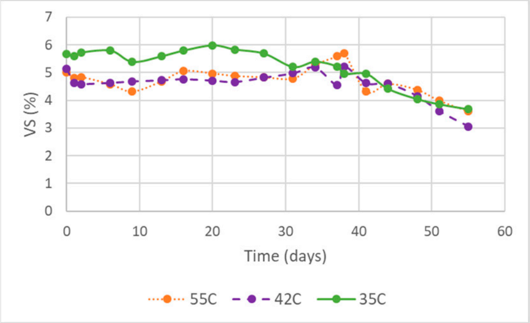

(**a**)

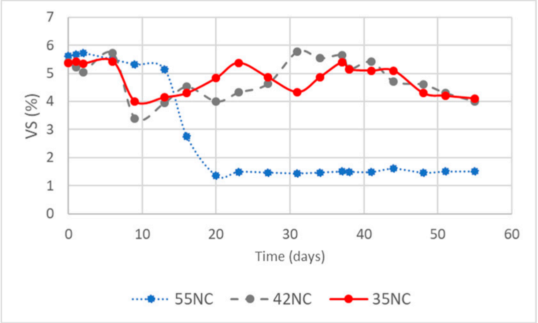

(**b**)

Figure S3. Evolution of volatile solids (VS, expressed as %) at thermophilic (55ºC), intermediate (42ºC) and mesophilic (35ºC) temperature: a) chrome-tanned (C); b) chromium-free (NC) leather shavings.

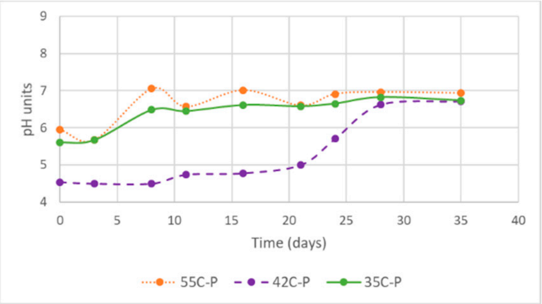

(**a**)

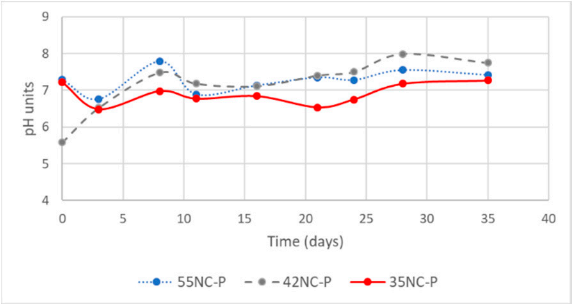

(**b**)

Figure S4. Evolution of pH at thermophilic (55ºC), intermediate (42ºC) and mesophilic (35ºC) temperature regimes: a) chrome-tanned (C) and b) chromium-free (NC) pretreated leather shavings.

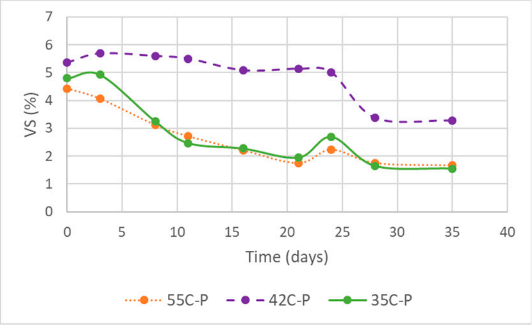

(**a**)

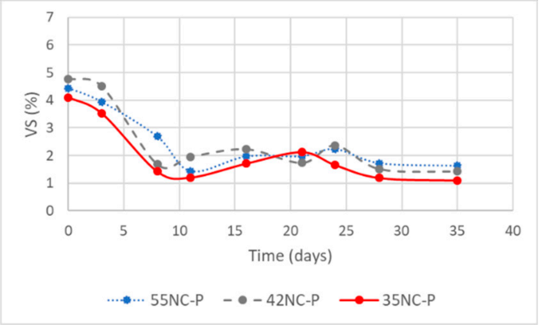

(**b**)

Figure S5. Evolution of volatile solids (VS, expressed as %) at thermophilic (55ºC), intermediate (42ºC) and mesophilic (35ºC) temperature regime: a) chrome-tanned (C) and b) chromium-free (NC) pretreated leather shavings.
